# Recyclable and Mendable Cellulose-Reinforced Composites Crosslinked with Diels–Alder Adducts

**DOI:** 10.3390/polym11010117

**Published:** 2019-01-11

**Authors:** KeumHwan Park, Cheolmin Shin, Ye-Seul Song, Hee-Jin Lee, Chiho Shin, Youngmin Kim

**Affiliations:** 1 Display Materials & Components Research Center, Korea Electronics Technology Institute, 25 Saenari-ro, Bundang-gu, Seongnam-si, Gyeonggi-do 13509, Korea; khpark@keti.re.kr (K.P.); bp53224@daum.net (Y.-S.S.); gmlwls5010@gmail.com (H.-J.L.); 2 Kwangsung Corporation Co., Ltd. 212-14, Neungan-gil, Songsan-myeon, Dangjin-Si, Chungcheongnam-do 31711, Korea; cmshin@belite.co (C.S.); chshin@belite.co (C.S.)

**Keywords:** cellulose, reinforced, composite, healing, recycling

## Abstract

Owing to their natural abundance and exceptional mechanical properties, cellulose fibers (CFs) have been used for reinforcing polymers. Despite these merits, dispersing hydrophilic CFs in a hydrophobic polymer matrix is challenging. To address this, an amphiphilic ammonium salt was employed as the dispersant for CFs in this study. The hydrophobic CFs were mixed with a healable polymer to produce CF-reinforced composites. As the thermosetting polymer was crosslinked with Diels–Alder (DA) adducts, it was mended and recycled via a retro DA reaction at 120 °C. Interestingly, the CF-reinforced polymer composites were mended and recycled as well. When 5 wt % of the hydrophobic CFs was added to the polymer, maximum tensile strength, elongation at break, Young’s modulus, and toughness increased by 70%, 183%, 75%, and 420%, respectively. After recycling, the CF-reinforced composites still featured better mechanical properties than recycled polymer.

## 1. Introduction

Cellulose fibers (CFs) have drawn much attention as composite materials due to their intrinsic advantages, such as natural abundance, low cost, and excellent mechanical properties [1]. As celluloses feature a linear chain in which glucose is linked through *β*-1,4-glucosidic bonds, they aggregate to form fibers through hydrogen bonding [2]. This provides CFs with a high elastic modulus of 138 GPa [3]. Although this hydrogen bonding provides CFs with excellent mechanical properties, it hampers CF dispersion in the hydrophobic polymer matrix in composites. To overcome this, celluloses have been modified using NIO_4_ [4,5], acid anhydride [6], carbonyl chloride [7,8], TEMPO/NaClO [9,10], deep-eutectic solvents [11,12], and silanes [13]. Although these methods are effective for the modification of CFs, they require multiple steps and the use of organic solvents. To reduce the cost of composite fabrication, a simple and cost-effective way to generate hydrophobic CFs is needed.

Polymers containing reversible bonds can mend damage when they are impacted, which ultimately extends their lifetime [14,15]. Polymers featuring disulfide bonds [16,17], dynamic urea bonds [18,19], π–π interaction [20], and hydrogen bonding [21,22,23] have been studied for application as self-healable materials. As the flow of polymers is the key factor in healing damage, a high temperature is required for healing to occur [24]. Although some polymers could mend under mild conditions, their poor mechanical properties needed to be improved for their practical application [16,17]. Recently, a polymer featuring dynamic urea bonds showed a high E-modulus of 0.34 GPa and a healing efficiency of 50% at 100 °C [25]. Additionally, Kim et al. reported that a disulfide-containing polymer with the excellent mechanical properties was mended at room temperature (RT) within 2 h [26]. Since Diels–Alder (DA) adducts were reversibly converted to their dienes and dienophiles through a retro DA reaction [27], they have also been employed as crosslinkers for self-healable polymers [28,29,30]. When furans and maleimides were employed as dienes and dienophiles, respectively, DA reaction and retro DA reaction occurred at around 50 and 120 °C, respectively. The healing efficiency of this combination reached 83% [31], implying that polymers with DA adducts are promising healable materials. To improve the mechanical properties of healable polymers, we fabricated CF-reinforced composites using thermosetting polymers with DA adducts as crosslinkers. The simple treatment of cationic surfactant made CFs hydrophobic, which enhanced the dispersion of the CFs in the healable polymers. The effect of the CFs on the mechanical properties of the composites was investigated. While mechanical properties of the composites with 1–5 wt % CFs increased, those of the composite with 7 wt % CFs decreased due to agglomeration of the CFs in the composite. Like unreinforced polymer, CF-reinforced polymer composites were healable via a retro DA reaction. Besides this, a retro DA reaction made thermosetting polymers and composites recyclable through dissolution processes.

## 2. Materials and Methods

### 2.1. Materials

Poly(propylene glycol) (*M*_n_ ~ 1000 g/mol), tetramethylammonium chloride (TM), tetrabutylammonium chloride (TB), hexadecyltrimethylammonium chloride (TH), propylene glycol monomethyl ether acetate (PGMEA), methyl ethyl ketone (MEK), dimethylformamide (DMF), and dibutyltin dilaurate were purchased from Sigma-Aldrich Korea Ltd, Yongin, Korea. Isophorone diisocyanate (IPDI), bis(3-ethyl-5-methyl-4-maleimidophenyl)methane (BMI), and furfurylamine were purchased from SEJINCI, Seoul, Korea. Cellulose was purchased from MOORIM P&P, Seoul, Korea. All chemicals were used as received, without purification. The furan diol **1** was synthesized by following the known method [32]. The poly(propylene glycol) used was designated as PPO-1000.

### 2.2. Preparation of the TH-Treated CFs

An aqueous suspension of CFs (166.6 g, 3.0 wt %) was diluted with deionized water (100 g) to produce a CF suspension with a solid content of 1% by weight. This was mixed with hexadecyltrimethylammonium chloride (5.0 g) and stirred at room temperature. After 30 min, hydrophobic CFs were isolated by filtration using a Buchner funnel, and dried in an oven (KIPAE ENT CO., LTD, Suwon, Korea).

### 2.3. Preparation of the F-PU Solution

The flask was charged with furan diol **1** (2.15 g, 10.0 mmol) and PPO-1000 (10.0 g, 10.0 mmol). To this mixture were added IPDI (4.5 g, 20.2 mmol) and PGMEA (16.6 g). A catalytic amount of dibutyltin dilaurate was added to the mixture at room temperature. The solution was heated at 60 °C for 3 h and cooled to room temperature to produce an F-PU solution with a solid content of 50% by weight.

### 2.4. Synthesis of the DA-PU

To the as-prepared F-PU in PGMEA (5.0 g) was added BMI (0.33 g). This mixture was heated at 60 °C. After 20 min, the solution was poured into a PTFE mold, followed by being kept in an oven. Oven temperature was increased from 40 to 80 °C slowly to produce a DA-PU pad.

### 2.5. Synthesis of the CF-Reinforced Composites

To the as-prepared F-PU in PGMEA (5.0 g) were added the TH-treated CFs. Once the cellulose fibers were dispersed in the F-PU solution, BMI (0.33 g) was added and stirred at 60 °C for 20 min. The solution was poured into a PTFE mold, followed by being kept in an oven. Oven temperature was increased from 40 to 80 °C slowly to produce CF-reinforced composites. The ratio of CF/DA-PU ranged from 1% to 7% by weight.

### 2.6. Healing Tests

The as-prepared DA-PU solution was poured on a glass substrate with dimensions of 5 cm × 5 cm. Then, it was spun at 500 rpm for 20 s to form a thin polymer layer on the glass substrate. This was kept at 80 °C overnight to produce a DA-PU layer on the glass substrate. The DA-PU layer was scratched by a cutter knife, then it was kept at 120 °C for 20 min for healing. The change in the scratch before and after thermal treatment was monitored by optical microscopy. For the healing test of a composite, a solution containing CF-reinforced composite with a CF content of 5 wt % was used.

### 2.7. Instrumentation

The proton NMR spectra were measured at 400 MHz on an NMR spectrometer equipped with Bruker Top Spin 3.2 software (AscendTM 400, Bruker, Madison, WI, USA) using DMSO-*d*_6_ as a solvent at room temperature. FTIR spectra in the range from 600 to 4000 cm^−1^ were measured on a Fourier transform infrared spectrophotometer (IRAffinity-1S, SHIMADZU, Kyoto, Japan), by placing samples on the surface of a ZnSe crystal using the attenuated total reflectance method. Surface profiles were measured by laser confocal microscopy (VK-9710K, Keyence, Osaka, Japan). The surfaces of the fractured samples were observed by scanning electron microscopy (SEM; JSM-6480, JEOL Ltd., Tokyo, Japan). Thermal stability of polymers, TH-treated CFs, and CF-reinforced composites were evaluated under N_2_ atmosphere by elevating the temperature from 23 to 400 °C at the rate of 10 °C/min using a differential thermal thermogravimetry analyzer (DTG-60H, Shimadzu, Japan). The molecular weight was obtained on an ACQUITY APC (Waters Corporation, Milford, MA, USA) equipped with a column (ACQUITY APC XT 2002.5 µm) by flowing a THF solution of a sample at the rate of 0.7 mL/min. After calibrating the column set using polystyrenes with molecular weights ranging from 1220 to 264,000 g/mol, the molecular weight of the sample was measured.

## 3. Results and Discussion

**Figure 1:**
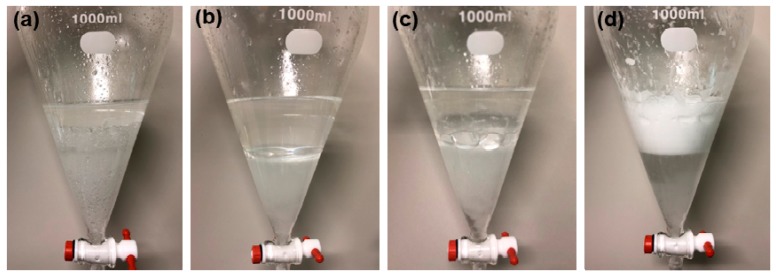
Cellulose fibers (CFs) in water/toluene solution after mixing. The CFs were treated with the following surfactants: (**a**) none, (**b**) tetramethylammonium chloride (TM), (**c**) tetrabutylammonium chloride (TB), and (**d**) hexadecyltrimethylammonium chloride (TH).

To make CFs hydrophobic, their aqueous solutions were treated with quaternary alkyl ammoniums (QAAs). The absorption of the QAAs on the surface of the CFs would alter the hydrophilicity of the CFs. We chose three types of QAAs (i.e., TM, TB, TH) to investigate the effect of the alkyl groups on the hydrophobicity of the CFs. The changes in the hydrophobicity of the QAA-treated CFs were readily observed by the extraction method using a water/toluene mixture. In this, the aqueous suspension of CFs was diluted to 1% using de-ionized water and stirred for 30 min. The solution was stirred by a magnetic stirrer at RT for 30 min. Then, toluene was added to the CF suspension to make the ratio of water/toluene one-to-one by weight. After the two layers were mixed in a separatory funnel, the mixture was allowed to stand for 30 min to check whether the CFs had been transferred to the organic layer (Figure 1). The CFs in the water phase were not transferred to the toluene phase when TM and TB were used as the dispersants. However, the use of TH made the CFs stay in the toluene phase instead of the water phase. The different behaviors of the dispersant-treated CFs may be attributed to the hydrophobicity and the shape of the dispersants. For example, the effect of cetyltrimethylammonium bromide on exfoliating graphite was reported previously [33]. Although TB and TH possess comparable carbon numbers, TB did not extract the CFs from the water phase to the toluene phase, unlike TH. This indicates that the bulky substituents of the butyl groups hampered interactions between the TB cations and the hydroxyl groups on the celluloses. In the case of TM, the smaller size of the substituent could lead to the interaction between the dispersants and the CFs, but the hydrophobicity of methyl groups was not large enough to extract the CFs in water by toluene.

**Scheme 1:**
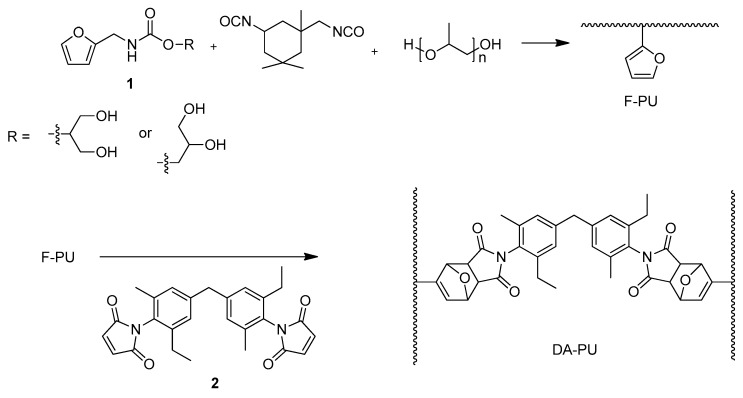
Synthesis of F-PU and DA-PU.

To fabricate healable composites using the TH-treated CFs, we synthesized polymers crosslinked by DA adducts. Furan diol **1** was readily synthesized by reacting glycerol carbonate and furfuryl amine at the ratio of 1:1 following the reported method. Then, a mixture of furan diol **1**, polyol (PPO-1000), and IPDI in PGMEA was heated at 60 °C for 3 h to produce F-PU, in which the furan groups were grafted onto the polyurethane backbones (Scheme 1). The completion of the reaction between the polyol and the isocyanate was indicated by the disappearance of the IR absorption at 2260 cm^−1^, corresponding to NCO stretching (Figure S1). The identity of F-PU was characterized by NMR spectroscopy. The appearance of the proton resonance at 4.8–4.9 ppm in the ^1^H-NMR spectrum showed that the hydroxyl group in the polyol had reacted with isocyanate to form a urethane bond. This was further confirmed by 2D COSY-NMR (Figure S2), in which there was a correlation between two protons at 4.86 and 1.20 ppm. In addition, the carbon resonance at 155.5 ppm further confirmed the presence of the urethane bond in the ^13^C-NMR spectrum. The weight average and number average molecular weights of F-PU were measured to be 40,302 and 34,270 g/mol, respectively. The polydispersity (*M*_w_/*M*_n_) was calculated to be 1.176.

**Figure 2:**
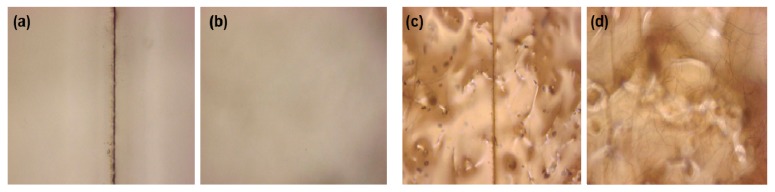
Optical microscopy images. The surface of DA-PU (**a**) before and (**b**) after the healing process. The surface of the composite with 5 wt % CFs (**c**) before and (**d**) after the healing process.

To synthesize polyurethane crosslinked through DA adducts, bismaleimide **2** was added to F-PU in PGMEA, and the solution was spin-coated on a glass substrate followed by drying at 80 °C overnight. When the solvent was slowly evaporated, DA adducts formed to produce a free-standing film of DA-PU. Owing to the crosslinking through DA adducts, thermosetting DA-PU was not dissolved in organic solvents such as MEK and DMF at RT. To investigate a retro DA reaction, DA-PU was immersed in DMSO-*d*_6_ and kept at 120 °C for 20 min. DA-PU powder was dissolved in DMSO-*d*_6_ over time to make the solution yellow in color (Figure S3). The deuterated solution was characterized by ^1^H-NMR spectroscopy. The ^1^H-NMR spectrum showed resonances at 6.21, 6.35, and 7.53 ppm, corresponding to protons of furan, indicating that the thermal treatment led to a retro DA reaction (Figure S4). The proton resonances at 7.21 ppm for ethylenic protons of maleimide confirmed the retro DA reaction as well. The de-crosslinking of a polymer was crucial for its healing ability because a soft polymer flowed readily and filled scratches. We performed healing tests with this compound (Figure 2). The surface of DA-PU was scratched by a cutter knife, then it was kept at 120 °C. After 20 min, the mark on the surface had completely disappeared and the polymer became yellow, indicating that a retro DA reaction had occurred.

**Figure 3:**
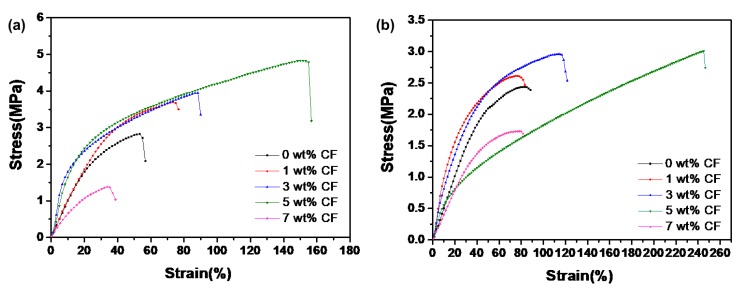
Stress-strain curves of CF-reinforced DA-PU composites (**a**) before and (**b**) after recycling.

Besides the self-healing ability, a retro DA reaction made the thermosetting polymers recyclable [34]. To this, the mixture of F-PU and bismaleimide **2** in PGMEA was poured into a PTFE mold with the size of 4 cm × 2 cm, and heated from 40 to 80 °C slowly to produce rectangular DA-PU samples. For recycling of DA-PU, it was dissolved in PGMEA. As the pad was not dissolved in PGMEA at RT, the solution was heated at 120 °C for 1 h. When the solution was clear, it was poured into the PTFE mold and maintained at temperatures in the range of 40 to 80 °C to produce pads of the same shape. Although there was a slight difference in color between the two pads, the thermosetting was successfully recycled. The thermal stability of the DA-PU was evaluated by increasing the temperature up to 400 °C by thermogravimetric analysis (TGA). The results showed that DA-PU was stable below 250 °C with little change in weight (Figure S5). As thermosetting DA-PU was mended and recycled at 120 °C, its decomposition was not a concern. These results were in good agreement with the thermal stability of other polymers crosslinked by DA adducts [32]. The mechanical properties of DA-PU were measured through a tensile test (Figure 3). A polymer sample with dimensions of 4 cm × 2 cm was extended along the *z*-axis until it was broken. The maximum stress and the elongation at break of the polymer were 2.83 MPa and 53%, respectively. Recycling through the dissolution process changed these properties of the recycled sample to 2.44 MPa and 81%. The change presumably stemmed from the different density in the crosslinking of the two samples due to the reversibility of the DA adducts. They were comparable to the maximum stress (2.4 MPa) and the elongation at break (88%) of the silicone-based polyurethane crosslinked with DA adducts in the previous report [34].

Encouraged by this, we mixed TH-treated CFs and DA-PU to improve the mechanical properties of DA-PU. To compare the dispersibility of TH-treated CFs with that of pristine CFs, they were mixed with a PGMEA solution of F-PU, at the CF/F-PU ratio of 100-to-5 by weight, and sonicated for 30 min. After the addition of bismaleimde **2** to both composite solutions, they were poured into PTFE molds and kept in an oven overnight. While the TH-treated CFs were well dispersed in the polymer, the pristine CFs aggregated in the matrix (Figure S6). This demonstrates that the hydrophobicity facilitated dispersion of the TH-treated CFs in the hydrophobic polymer matrix. Despite this, aggregation of the TH-treated CFs was monitored when the CF-to-polymer ratio was 7% by weight. To investigate the effect of the TH-treated CFs on the mechanical properties, composites with CF/DA-PU ratios ranging from 1% to 7% by weight were prepared. The mechanical properties of the composites are summarized in Table 1. 

**Figure 4:**
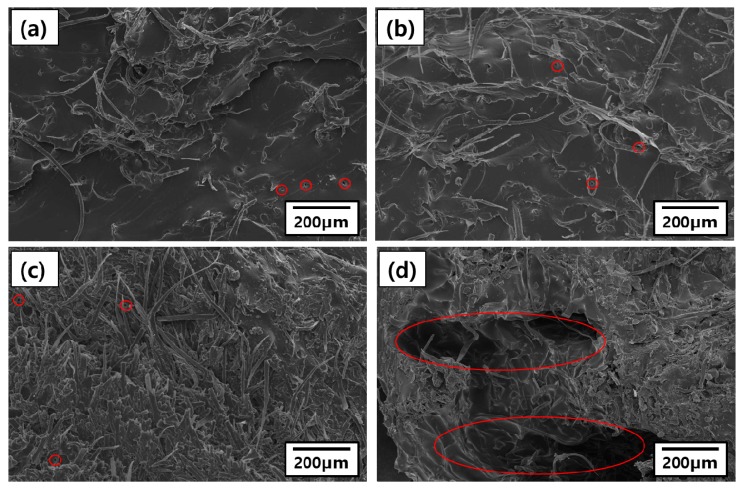
SEM images of the surfaces of fractures in the composites after the tensile tests. Composites with a CF content of (**a**) 1 wt %, (**b**) 3 wt %, (**c**) 5 wt %, and (**d**) 7 wt %. Red circles indicate holes generated by the pullout of the CFs.

The stress-strain curves show that mechanical properties were improved as the CF content in the thermosetting composites increased (Figure 3). When the CFs were mixed in the polymer by 5 wt %, maximum tensile strength, elongation at break, Young’s modulus, and toughness increased by 70%, 183%, 75%, and 420%, respectively, in comparison to unreinforced DA-PU. This demonstrates that the addition of CFs made the composite tough. Even though the increase in both modulus and elongation of the composites was unusual, some composites with carbon nanotubes [35,36,37] and graphene oxide [38] have shown similar results. The Young’s modulus was higher for the composite with a CF content of 3 wt % than that with a CF content of 5 wt % because the increased modulus by CFs was compromised by TH, which acted as a plasticizer in composites [39]. Composites also mended scratches like DA-PU under the same conditions, indicating that the improved mechanical properties did not deteriorate the healing efficiency of the polymer (Figure 2). Well-dispersed CFs in the DA-PU polymer matrix were also observed by optical microscopy after the healing test. However, the mechanical properties of the composite with a CF content of 7 wt % were worse than those of DA-PU due to poor dispersion of the CFs in the polymer (Figure 3). Once the CFs aggregated to become bundles in the composites, they served as stress concentrators, which induced pull-out failure. The surfaces of the fractures in the composites after the tensile tests were analyzed by scanning electron microscopy (Figure 4). While the composites with a CF content of 1–5 wt % showed small holes (ca. 10 µm), those with a CF content of 7 wt % featured large holes (>ca. 100 µm). As the diameter of the CFs was ca. 10 µm, the large holes stemmed from the pullout of the CF aggregates. This implied that dispersion of the CFs plays a key role in enhancing the mechanical properties of composites [35].

We recycled the CF-reinforced thermosetting polymer composites by dissolving them in PGMEA at 120 °C, followed by pouring in a PTFE mold. The mechanical properties of the recycled ones were measured and are summarized in Table 2. The stress-strain curves of recycled composites showed that tensile strength and modulus were decreased, with a concomitant increase in elongation. This indicated that crosslinking density of the composites was reduced after recycling because DA adducts were reversibly converted to their dienes and dienophiles at high temperature. The toughness of composites was rather similar before and after recycling. When the composite with a CF content of 5 wt % was recycled, tensile strength, elongation at break, Young’s modulus, and toughness increased by 23%, 200%, 42%, and 224% in comparison to the recycled DA-PU polymer. Unlike other composites, the toughness of the composite with a CF content of 7 wt % was improved after recycling. This was probably due to the increased dispersion of CFs in the composite solution at high temperature during recycling.

## 4. Conclusions

Hydrophobic CFs were not only dispersed in hydrophobic polymers but also acted as both fillers and plasticizers, which improved the mechanical properties of composites. The combined enhancement in Young’s modulus and elongation realized that toughness of the composites increased in proportion to the amount of the dispersed CFs compared to the unreinforced polymer. The incorporation of the hydrophobic CFs in thermosetting, crosslinked by DA adducts, resulted in highly-durable mendable composites without deteriorating healing ability. In addition, thermosetting composites were recyclable via a retro DA reaction, which is not possible for conventional thermosetting crosslinked by irreversible bonds.

## Figures and Tables

**Table 1:** Mechanical properties of polymer and composites.

CF Content	Tensile Strength (MPa)	Elongation at Break	Young’s Modulus (MPa)	Toughness (MJ/m^3^)
0 wt %	2.83 ± 0.06	53.55 ± 3.35	10.10 ± 0.32	1.09 ± 0.02
1 wt %	3.70 ± 0.24	73.33 ± 8.63	10.80 ± 0.87	1.96 ± 0.04
3 wt %	3.95 ± 0.31	88.33 ± 6.20	23.80 ± 2.67	2.62 ± 0.02
5 wt %	4.83 ± 0.82	151.57 ± 28.01	17.74 ± 2.29	5.67 ± 0.06
7 wt %	1.38 ± 0.25	36.66 ± 10.42	7.21 ± 1.60	0.35 ± 0.29

**Table 2:** Mechanical properties of polymer and composites after recycling.

CF Content	Tensile Strength (MPa)	Elongation at Break	Young’s Modulus (MPa)	Toughness (MJ/m^3^)
0 wt %	2.44 ± 0.09	81.67 ± 9.36	4.61 ± 0.25	1.48 ± 0.03
1 wt %	2.61 ± 0.20	76.66 ±17.14	9.83 ± 1.50	1.63 ± 0.06
3 wt %	2.96 ± 0.37	113.33 ± 12.73	9.14 ± 1.74	2.70 ± 0.04
5 wt %	3.01 ± 0.81	245.00 ± 44.33	6.57 ± 1.36	4.80 ± 0.08
7 wt %	1.73 ± 0.44	78.33 ± 42.30	4.00 ± 1.15	0.98 ± 0.71

## Supplementary Materials

The following are available online at http://www.mdpi.com/2073-4360/11/1/117/s1, Figure S1. FTIR spectrum of F-PU. Figure S2. 2D COSY NMR of polymer F-PU. There was a correlation between the proton (a) next to the urethane bond and the protons (b) on the methyl group. Figure S3. DA-PU in the NMR tube (left) before and (right) after being heated at 120 °C for 20 min. Figure S4. ^1^H-NMR spectrum of DA-PU in DMSO-*d*_6_ ranging from 6.0 ppm to 8.0 ppm after heat treatment. Figure S5. TGA graphs over temperature for (a) TH-treated CFs, (b) DA-PU and (c) the CF-reinforced composite. Figure S6. CF-reinforced polymer composites with a CF content of 5 wt %. The composites were prepared using (left) the TH-treated CFs and (right) the pristine CFs.

## References

[1] Lee K.-Y., Aitomäki Y., Berglund L.A., Oksman K., Bismarck A. (2014). On the use of nanocellulose as reinforcement in polymer matrix composites. Compos. Sci. Technol..

[2] Cazón P., Vázquez M., Velazquez G. (2018). Novel composite films based on cellulose reinforced with chitosan and polyvinyl alcohol: Effect on mechanical properties and water vapour permeability. Polym. Test..

[3] Sato A., Kabusaki D., Okumura H., Nakatani T., Nakatsubo F., Yano H. (2016). Surface modification of cellulose nanofibers with alkenyl succinic anhydride for high-density polyethylene reinforcement. Compos. Part A.

[4] Liimatainen H., Visanko M., Sirviö J.A., Hormi O.E.O., Niinimaki J. (2012). Enhancement of the Nanofibrillation of Wood Cellulose through Sequential Periodate–Chlorite Oxidation. Biomacromolecules.

[5] Sirvio J., Hyvakko U., Liimatainen H., Niinimaki J., Hormi O. (2011). Periodate oxidation of cellulose at elevated temperatures using metal salts as cellulose activators. Carbohydr. Polym..

[6] Pandey J.K., Chu W.S., Kim C.S., Lee C.S., Ahn S.H. (2009). Bio-nano reinforcement of environmentally degradable polymer matrix by cellulose whiskers from grass. Compos. Part B.

[7] Freire C.S.R., Silvestre A.J.D., Neto C.P., Belgacem M.N., Gandini A. (2006). Controlled heterogeneous modification of cellulose fibers with fatty acids: Effect of reaction conditions on the extent of esterification and fiber properties. J. Appl. Polym. Sci..

[8] Freire C.S.R., Silvestre A.J.D., Pascoal Neto C., Rocha R.M.A. (2005). An Efficient Method for Determination of the Degree of Substitution of Cellulose Esters of Long Chain Aliphatic Acids. Cellulose.

[9] Habibi Y., Chanzy H., Vignon M.R. (2006). TEMPO-mediated surface oxidation of cellulose whiskers. Cellulose.

[10] Saito T., Nishiyama Y., Putaux J.-L., Vignon M., Isogai A. (2006). Homogeneous Suspensions of Individualized Microfibrils from TEMPO-Catalyzed Oxidation of Native Cellulose. Biomacromolecules.

[11] Hosseinmardi A., Annamalai P.K., Wang L., Martin D., Amiralian N. (2017). Reinforcement of natural rubber latex using lignocellulosic nanofibers isolated from spinifex grass. Nanoscale.

[12] Sirviö J.A., Visanko M., Liimatainen H. (2015). Deep eutectic solvent system based on choline chloride-urea as a pre-treatment for nanofibrillation of wood cellulose. Green Chem..

[13] Fu J., Zhang J., Song X., Zarrin H., Tian X., Qiao J., Rasen L., Li K., Chen Z. (2016). A flexible solid-state electrolyte for wide-scale integration of rechargeable zinc–air batteries. Energy Environ. Sci..

[14] Yang Y., Ding X., Urban M.W. (2015). Chemical and physical aspects of self-healing materials. Prog. Polym. Sci..

[15] An S.Y., Arunbabu D., Noh S.M., Song Y.K., Oh J.K. (2015). Recent strategies to develop self-healable crosslinked polymeric networks. Chem. Commun..

[16] Rekondo A., Martin R., Ruiz de Luzuriaga A., Cabañero G., Grande H.J., Odriozola I. (2014). Catalyst-free room-temperature self-healing elastomers based on aromatic disulfide metathesis. Mater. Horiz..

[17] Canadell J., Goossens H., Klumperman B. (2011). Self-Healing Materials Based on Disulfide Links. Macromolecules.

[18] Ying H., Zhang Y., Cheng J. (2014). Dynamic urea bond for the design of reversible and self-healing polymers. Nat. Commun..

[19] Park J.I., Choe A., Kim M.P., Ko H., Lee T.H., Noh S.M., Kim J.C., Cheong I.W. (2018). Water-adaptive and repeatable self-healing polymers bearing bulky urea bonds. Polym. Chem..

[20] Burattini S., Colquhoun H.M., Fox J.D., Friedmann D., Greenland B.W., Harris P.J.F., Hayes W., Mackay M.E., Rowan S.J. (2009). A self-repairing, supramolecular polymer system: Healability as a consequence of donor–acceptor π–π stacking interactions. Chem. Commun..

[21] Zhang D.-D., Ruan Y.-B., Zhang B.-Q., Qiao X., Deng G., Chen Y., Liu C.-Y. (2017). A self-healing PDMS elastomer based on acylhydrazone groups and the role of hydrogen bonds. Polymer.

[22] Montarnal D., Cordier P., Soulié-Ziakovic C., Tournilhac F., Leibler L. (2008). Synthesis of self-healing supramolecular rubbers from fatty acid derivatives, diethylene triamine, and urea. J. Polym. Sci. Part A Polym. Chem..

[23] Folmer B.J.B., Sijbesma R.P., Versteegen R.M., van der Rijt J.A.J., Meijer E.W. (2000). Supramolecular Polymer Materials: Chain Extension of Telechelic Polymers Using a Reactive Hydrogen-Bonding Synthon. Adv. Mater..

[24] Garcia S.J. (2014). Effect of polymer architecture on the intrinsic self-healing character of polymers. Eur. Polym. J..

[25] Zechel S., Geitner R., Abend M., Siegmann M., Enke M., Kuhl N., Klein M., Vitz J., Gräfe S., Dietzek B. (2017). Intrinsic self-healing polymers with a high E-modulus based on dynamic reversible urea bonds. NPG Asia Mater..

[26] Kim S.-M., Jeon H., Shin S.-H., Park S.-A., Jegal J., Hwang S.Y., Oh D.X., Park J. (2018). Superior Toughness and Fast Self-Healing at Room Temperature Engineered by Transparent Elastomers. Adv. Mater..

[27] Liu Y.-L., Chuo T.-W. (2013). Self-healing polymers based on thermally reversible Diels–Alder chemistry. Polym. Chem..

[28] Chen X., Dam M.A., Ono K., Mal A., Shen H., Nutt S.R., Sheran K., Wudl F. (2002). A Thermally Re-mendable Cross-Linked Polymeric Material. Science.

[29] Lee D.H., Heo G., Pyo K.-H., Kim Y., Kim J.-W. (2016). Mechanically Robust and Healable Transparent Electrode Fabricated via Vapor-Assisted Solution Process. ACS Appl. Mater. Interfaces.

[30] Heo G., Pyo K.-H., Lee D.H., Kim Y., Kim J.-W. (2016). Critical Role of Diels–Adler Adducts to Realise Stretchable Transparent Electrodes Based on Silver Nanowires and Silicone Elastomer. Sci. Rep..

[31] Du P., Wu M., Liu X., Zheng Z., Wang X., Sun P., Joncheray T., Zhang Y. (2014). Synthesis of linear polyurethane bearing pendant furan and cross-linked healable polyurethane containing Diels–Alder bonds. New J. Chem..

[32] Pyo K.-H., Lee D.H., Kim Y., Kim J.-W. (2016). Extremely rapid and simple healing of a transparent conductor based on Ag nanowires and polyurethane with a Diels–Alder network. J. Mater. Chem. C.

[33] Vadukumpully S., Paul J., Valiyaveettil S. (2009). Cationic surfactant mediated exfoliation of graphite into graphene flakes. Carbon.

[34] Kim J.-W., Lee D.H., Jeon H.-J., Jang S.I., Cho H.M., Kim Y. (2018). Recyclable thermosetting thermal pad using silicone-based polyurethane crosslinked by Diels-Alder adduct. Appl. Surf. Sci..

[35] Blond D., Barron V., Ruether M., Ryan K.P., Nicolosi V., Blau W.J., Coleman J.N. (2006). Enhancement of Modulus, Strength, and Toughness in Poly(methyl methacrylate)-Based Composites by the Incorporation of Poly(methyl methacrylate)-Functionalized Nanotubes. Adv. Funct. Mater..

[36] Yang B.-X., Shi J.-H., Pramoda K.P., Goh S.H. (2008). Enhancement of the mechanical properties of polypropylene using polypropylene-grafted multiwalled carbon nanotubes. Compos. Sci. Technol..

[37] Yang B.-X., Shi J.-H., Pramoda K.P., Goh S.H. (2007). Enhancement of stiffness, strength, ductility and toughness of poly(ethylene oxide) using phenoxy-grafted multiwalled carbon nanotubes. Nanotechnology.

[38] Zhao Y., Lu J., Liu X., Wang Y., Lin J., Peng N., Li J., Zhao F. (2016). Performance enhancement of polyvinyl chloride ultrafiltration membrane modified with graphene oxide. J. Colloid Interface Sci..

[39] Zou H., Xu W., Zhang Q., Fu Q. (2006). Effect of alkylammonium salt on the dispersion and properties of Poly(p-phenylene sulfide)/clay nanocomposites via melt intercalation. J. Appl. Polym. Sci..

