# Supplementary material for: Recyclable and Mendable Cellulose-Reinforced Composites Crosslinked with Diels–Alder Adducts
Source: Polymers (Basel). 2019 Jan 11;11(1):117. doi: 10.3390/polym11010117 (PMC6401934; doi:10.3390/polym11010117)
Supplement: Supplementary file 1 [file polymers-11-00117-s001.pdf]

Supplementary Materials

Recyclable and mendable cellulose-reinforced composites crosslinked with Diels-Alder adducts

KeumHwan Park [1], Cheolmin Shin [2], Ye-Seul Song [1], Hee-Jin Lee [1], Chiho Shin [2] and Youngmin Kim[1,*]

[1] Display Materials & Components Research Center, Korea Electronics Technology Institute, 25 Saenari-ro, Bundang-gu, Seongnam-si, Gyeonggi-do 13509, Korea
[2] Kwangsung Corporation Co., Ltd. 212-14, Neungan-gil, Songsan-myeon, Dangjin-Si, Chungcheongnam-do 31711, Korea
* Correspondence: ymkim109f@gmail.com; Tel.: +82 31 789 7432

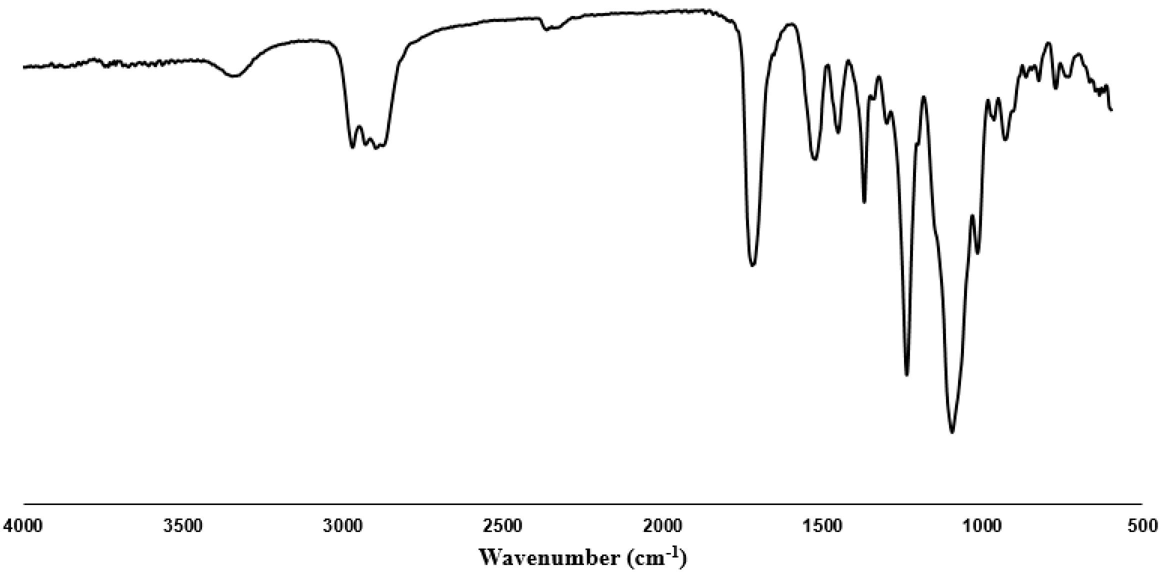

Figure S1. FTIR spectrum of F-PU.

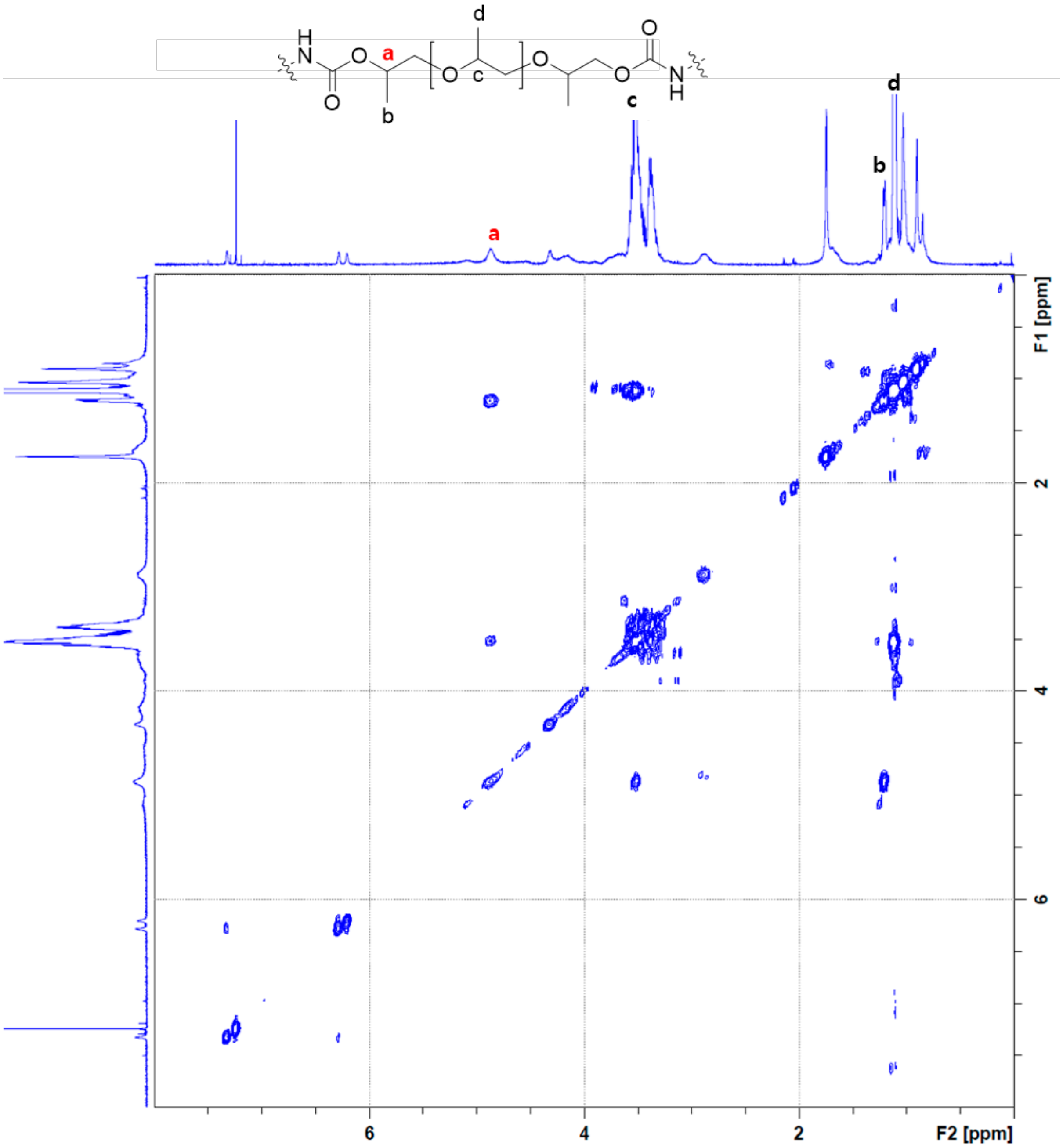

Figure S2. 2D COSY NMR of polymer F-PU. There was a correlation between the proton (a) next to the urethane bond and the protons (b) on the methyl group.

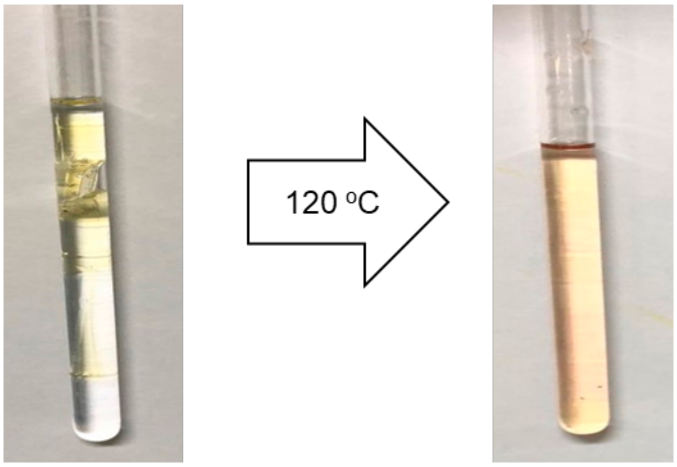

Figure S3. DA-PU in the NMR tube (left) before and (right) after being heated at 120 °C for 20 minutes.

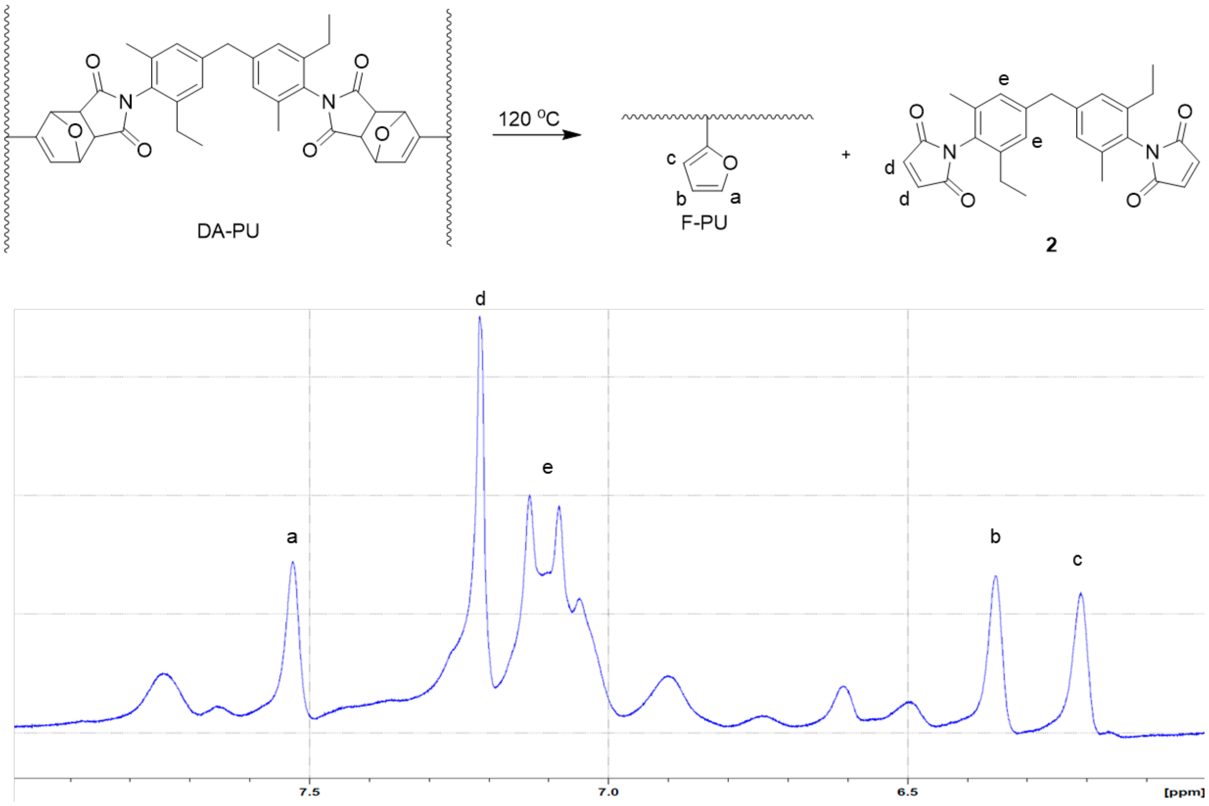

Figure S4. ^{1}H-NMR spectrum of DA-PU in DMSO-d_6 ranging from 6.0 ppm to 8.0 ppm after heat treatment.

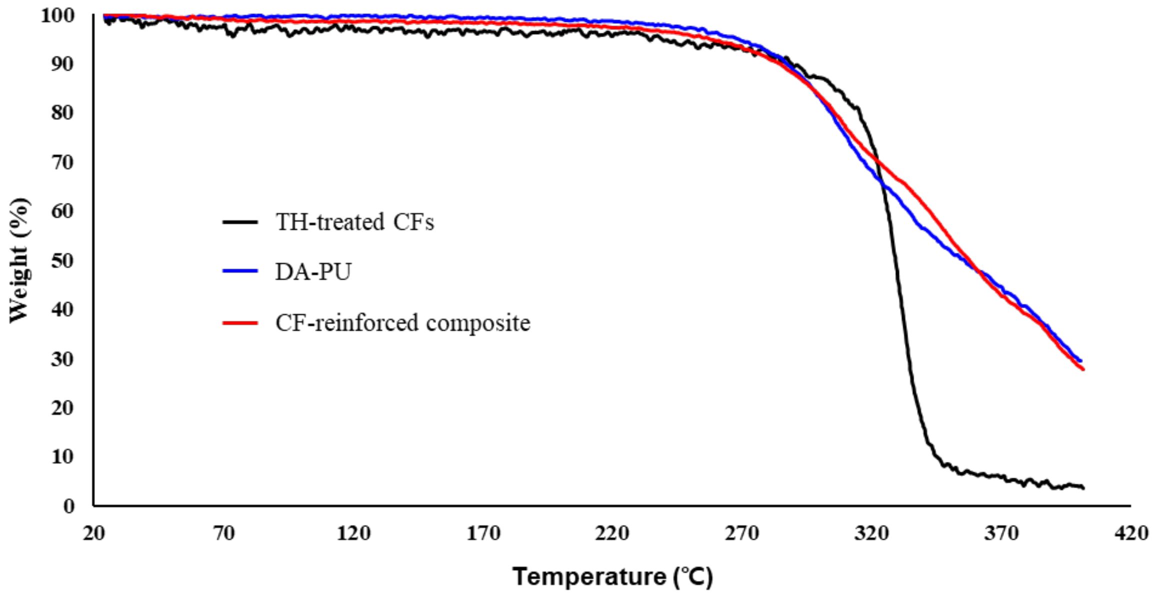

Figure S5. TGA graphs over temperature for (a) TH-treated CFs, (b) DA-PU and (c) the CF-reinforced composite.

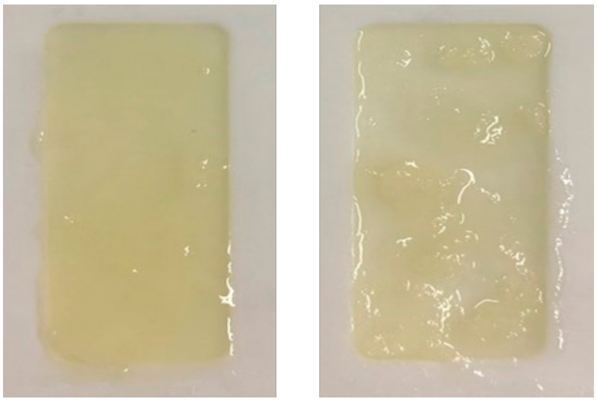

Figure S6. CF-reinforced polymer composites with a CF content of 5 wt%. The composites were prepared using (left) the TH-treated CFs and (right) the pristine CFs.
